# Unraveling the Impact of Malaria Exposure Before Birth

**DOI:** 10.1371/journal.pmed.1000117

**Published:** 2009-07-28

**Authors:** Lars Hviid

**Affiliations:** Centre for Medical Parasitology at University of Copenhagen and Copenhagen University Hospital (Rigshospitalet), Copenhagen, Denmark

## Abstract

Lars Hviid discusses a research article in *PLoS Medicine* that explores whether prenatal exposure to malaria is associated with increased susceptibility to malarial infection and anemia in infancy.

**Linked Research Article**

This Perspective discusses the following new study published in *PLoS Medicine*:

Malhotra I, Dent A, Mungai P, Wamachi A, Ouma JH, Narum DL, Muchiri E, Tisch DJ, King CL (2009) Can prenatal malaria exposure produce an immune tolerant phenotype?: A prospective birth cohort study in Kenya. PLoS Med 6(7): e1000116. doi:10.1371/journal.pmed.1000116

In a prospective cohort study of newborns residing in a malaria holoendemic area of Kenya, Christopher King and colleagues find a subset of children born to malaria-infected women who acquire a tolerant phenotype, which persists into childhood and is associated with increased susceptibility to malarial infection and anemia.

There are an estimated 250 million cases of *Plasmodium falciparum* malaria every year, which cost 1–2 million lives. The majority of serious and fatal cases occur among children below the age of five years, as substantial protective immunity is gradually acquired following repeated infections. Adolescents and adults therefore rarely suffer clinical episodes of malaria.

## Pregnancy-Associated Malaria

Nevertheless, women in such areas again become highly vulnerable to infection by *P. falciparum* parasites when they get pregnant, especially during a first pregnancy. This pregnancy-associated reappearance of susceptibility is related to the ability of the parasites to escape pre-existing clinical immunity by expressing particular pregnancy-specific antigens on the surface of infected erythrocytes [1]. These antigens, which appear largely synonymous with the VAR2CSA member of the PfEMP1 protein family [2], allow infected erythrocytes to accumulate on the maternal side of the placenta. The parasites often survive pre-existing immunity because VAR2CSA expression appears confined to placenta-sequestering parasites, which means that many pregnant women—particularly primigravidae—do not possess adequate VAR2CSA-specific immunity to clear them.

## Prenatal Exposure to Malaria Parasites

Placental parasitemia is associated with maternal anemia, prematurity, low birth weight, and excess perinatal morbidity and mortality [3]. An additional, but much less studied, consequence of placental parasitemia is that many newborns are born with immune systems that are already primed because they have been exposed to *P. falciparum* antigens in utero. Prenatal exposure can result in fetal acquisition of parasite-specific antibody and cytokine responses that can be measured in the infant's lymphocytes at delivery, but may also lead to tolerance and immunological anergy at subsequent re-exposure [4].

The time to first parasitemia is generally shorter in offspring of mothers with placental *P. falciparum* infection at delivery than in offspring of mothers without. This discrepancy is often interpreted as the consequence of impaired acquisition of protective immunity in children exposed to antigen while in the womb. However, in all likelihood a multitude of factors contribute and interact in complex ways, which are rarely considered in much detail.

## Does Prenatal Exposure Impair Acquisition of Protective Immunity?

In this week's *PLoS Medicine*, Christopher King and colleagues [5] report results from a study designed to directly address the hypothesis that prenatal exposure to parasite antigens directly affects the risk of malaria in infancy. The authors recruited 586 pregnant women and their newborn children from an area of stable transmission of *P. falciparum* parasites in coastal Kenya. Venous and placental blood from the mothers as well as cord blood from the babies was examined by microscopy and PCR for presence of parasites to evaluate prenatal exposure to parasite antigens. The authors then tested the parasite antigen-specific immune reactivity in the offspring at delivery and every six months thereafter for the first three years of life. The antigens examined were parasite proteins involved in merozoite invasion of erythrocytes, targets of protective immunity, and vaccine candidates. Antigen-induced responses included lymphocyte proliferation and cytokine production. Furthermore, the authors measured plasma levels of IgG with specificity for some of the antigens used in the in vitro assays of cellular immunity.

Once all data were collected, the authors compared the results in three sub-groups of the children. The first of these was composed of the 246 “sensitized” children, where cytokine responses (other than IL-10) could be detected in antigen-stimulated cord blood cultures. The second sub-group included the 120 “not sensitized” children, whose cord blood cells did not produce cytokine responses despite parasitological evidence of in utero exposure. Finally, the third sub-group consisted of the 220 “not exposed” children, where no antigen-induced cord lymphocyte cytokines were detected, but where parasitological evidence of prenatal exposure could not be obtained.

The “not sensitized” children who appeared to have been tolerized to *P. falciparum* antigens before birth were approximately 40% more likely to become infected during the follow-up period than either sensitized or unexposed children. In contrast to earlier studies 6,7, maternal parity did not influence the risk of infection in the children. Parasitemias tended to be low, and not much different between groups. Nevertheless, the putatively tolerized children were more anemic than the other children. In addition, lymphocytes from the “not sensitized” children were less likely to produce cytokines such as IFN-γ and IL-2 and more likely to produce IL-10 in response to antigenic stimulation, particularly in the second half of the follow-up period. Similar and high plasma levels of malaria antigen-specific IgG were detected in all newborns (due to passive transfer of maternal IgG across the placenta). As expected, these levels fell to very low levels in the second half of the first year, and then slowly increased during the second year as the children started to acquire immunity to the parasites. There were no obvious differences between the groups of children with respect to acquisition of parasite-specific antibodies.

## Where From Here?

The authors conclude that their data show that a sizeable proportion of children in endemic areas are immunologically disadvantaged at birth as a consequence of being exposed to *P. falciparum* antigens before birth, and that this is of clinical importance because it increases their risk of parasitemia in the first few years of life. If this is indeed the case, it is of obvious importance to clinicians and health policy makers. However, there are issues that require further study and consideration by scientists in this field. First of all, it is intuitively surprising how such a disadvantage can persist despite the expected negative selection pressure against it. Secondly, it should be considered whether below average, Th1-type, “pro-inflammatory” cellular immune responses and low-level parasitemia are in fact adequate indicators of impaired clinical immunity, when several studies have shown that asymptomatic infection actually protects against clinical diseases episodes, and that eradication of such infections can be detrimental [8]–[10]. Thirdly, only parasitemia detectable at parturition was considered. However, the prevalence of placental infection peaks in the second trimester, and undoubtedly many infections with the potential to affect the immune system of the fetus have been resolved well before delivery [11]. Fourthly, the authors chose to study cytokine responses to “classical” parasite antigens rather than the antibody responses to the so-called variant surface antigens, which are important determinants of clinical immunity [12]. Finally, there are issues related to anti-malarial drug use and HIV infection that remain unresolved after the present study.

In conclusion, the study by King et al. adds significantly to our understanding of prenatal exposure to *P. falciparum* antigens. Hopefully, the study will inspire scientists in the field to study this complex and clinically significant issue further.
